# Natural Compounds as Potential Anticancer, Anti-Inflammatory, and Antioxidant Agents in Medicine

**DOI:** 10.3390/ph19040531

**Published:** 2026-03-25

**Authors:** Justyna Stefanowicz-Hajduk

**Affiliations:** Department of Biology and Pharmaceutical Botany, Medical University of Gdańsk, 80-416 Gdańsk, Poland; justyna.stefanowicz-hajduk@gumed.edu.pl

Natural compounds play a significant role in the prevention and treatment of human diseases, becoming the basis for traditional remedies and modern pharmaceuticals. Due to their generally favorable safety profiles and wide range of therapeutic properties, more and more attention is being paid to these compounds, which are mainly derived from plants, marine species and microorganisms. Of particular interest are compounds with anticancer, antioxidant, and anti-inflammatory activities, which are promising agents with therapeutic potential.

Chronic inflammatory processes can lead to the development of cancer, which is often associated with oxidative stress. An excess of free radicals in the body is the result of an imbalance between the production of reactive oxygen species (ROS) and antioxidant system efficiency. Cancer development occurs at different rates and may lead to tissue invasion and disruption of normal organ function. Anticancer therapy often involves surgical resection of the tumor, as well as the use of chemotherapy, radiotherapy, and immunotherapy. However, these methods may prove insufficient to prevent further progression of the disease, which is why natural substances with multi-directional action are still being sought. It is well known that natural compounds belonging to various chemical groups such as flavonoids, phenolic acids, carotenoids, steroidal compounds, alkaloids, and terpenoids can effectively support the body’s antioxidant defense and modulate key molecular pathways involved in redox regulation, inflammation process, cell proliferation, and apoptosis. Hence, they can contribute to reducing the progress of lifestyle diseases and participate in their prevention.

This Special Issue focuses on research of natural compounds derived from plants (Contributions 1–4) and animals (Contribution 5) with anticancer (Contributions 1–3,5), anti-inflammatory (Contributions 3,4), and antioxidant properties (Contributions 2–4). It also includes one review that provides a comprehensive overview of the anticancer potential of *Sesbania* species and their main secondary metabolites (Contribution 6). 

Cancer remains one of the most significant challenges to global public health in the twenty-first century. The main causes of its development are a diet low in vegetables, sedentary lifestyle, smoking, age, genetic predispositions, and infections. The progressive aging of the population currently has a negative impact on the increased incidence of gastrointestinal, lung, and gynecological tumors. One such example is colon, stomach, and pancreatic cancer. Anifowose et al. proposed a potential use of *Aristolochia ringens* Vahl root extract in the treatment of colon adenocarcinoma (Contribution 1). The methanol extract was active on human colorectal Caco-2 and HT-29 cells with IC_50_ values of 26.61 ± 0.86 µg/mL and 32.89 ± 3.07 µg/mL, respectively. In Caco-2 cells, *A. ringens* extract induced cell cycle arrest in the G1 phase and apoptosis through the depolarization of the mitochondrial membrane. The presence of alkaloids, sesquiterpenes/diterpenes, and steroidal compounds with similarity to estra-5(10)-en-3-one-17-ol acetate or triterpenoid compound similar to ginsenol, detected in preliminary analysis, may be responsible for biological activity. Thus, further studies should evaluate and focus on the bioactivity-guided fractionation and identification of the cytotoxic secondary metabolites. 

Plant extracts contain a wealth of active compounds which can exhibit more potent anticancer, anti-inflammatory, and antioxidant effects than isolated secondary metabolites. This is often due to the synergistic effects of the active compounds, even if they do not dominate in the fraction being studied. To obtain potentially therapeutic plant extracts, a number of conventional solvents are used, such as ethanol, water, methanol, diethyl ether, or ethyl acetate. The use of a specific solvent determines the qualitative and quantitative composition of the obtained extracts, which results in their biological effect. This relationship has been demonstrated in another study, in which *Coleus aromaticus* Benth. extracts were tested against human colon cancer HCT-116 and gastric cancer AGS cells (Contribution 2). In this work, three different extracts, namely ethanol, ethanol/water, and juice, showed different activity towards the cancer cells. The most active was the ethanol extract, with IC_50_ values of 4.94 ± 0.48 and 24.99 ± 1.80 µg/mL on AGS and HCT-116 cells, respectively. Interestingly, the highest antioxidant potential was found in the ethanol extract, which had IC_50_ values of 13.34 ± 0.11 (ABTS), 22.90 ± 1.30 (DPPH), and 290.17 ± 4.23 µg/mL (molybdenum reducing power). Phytochemical analysis revealed that the highest amounts of phenolic and flavonoid compounds were found in the ethanol extract, with rosmarinic acid, caffeic acid, gallic acid, and luteolin. Moreover, carvacrol was found to be most abundant in the ethanol extract. Variations in (poly)phenolics and terpenes composition in the tested plant extracts determine their anticancer and antioxidant effects. Due to its ability to fight free radicals and induce tumor cell death, this plant has therapeutic potential, both in cancer prevention and treatment. 

Another example of synergistic effect between different plant compounds is study on chia seeds, which demonstrated anticancer, anti-angiogenic, antiproliferative, anti-inflammatory, and antioxidant effects (Contribution 3). Ali et al. tested chia (*Salvia hispanica* L.) seed extracts prepared with ether and 70% ethanol on lung cancer in both in vitro and in vivo models. The extracts showed significant cytotoxicity on A549 cell line with IC_50_ below 20 µg/mL. The effect was comparable to a well-known cytostatic—doxycycline. In the in vivo study, the chia extracts significantly reduced the relative weight of the lung in comparison to the cancer control. Furthermore, both chia extracts showed an anti-angiogenic effect, resulting in a significant reduction in vascular epithelial growth factor (VEGF), the angiogenic biomarker, and anti-inflammatory activity through reducing pro-inflammation interleukins IL-6 and IL-1β levels. Both extracts had also antioxidant potential by influencing on antioxidant enzymes—glutathione reductase (GR), glutathione S transferase (GST), and glutathione peroxidase (GPx)—in the lung cancer model. The main secondary metabolites that could be responsible for such biological and pharmacological effects of chia seeds were caffeic acid, vanillic acid hexoside, kaempferol-3-*O*-glucuronide, taxifolin, β-sitosterol, linolenic acid, diterpenes (trijugunone C, tanshinon), and alkaloid (jatrorrhizine).

In modern medicine, active plant substances are often combined with cytostatic drugs to enhance the therapeutic effect and limit side effects. Such a model was proposed by Guo et al. (Contribution 5), who studied the effects of using a novel combination of drugs in the treatment of pancreatic cancer. This type of cancer is often a lethal disease with an aggressive tumor biology and non-specific symptoms, and is predicted to become the second leading cause of cancer death by 2030. Treatment involves chemotherapy and surgical resection. However, only a small percentage of patients qualify for surgery. Thus, novel therapy in combination with well-known cytostatics is urgently needed [1]. Guo et al. demonstrated the mechanisms of action of the Chansu injection (CSI) derived from the secretion of the skin and parotoid glands of toad *Bufo gargarizans* Cantor. This formulation has been approved by the CFDA (China Food and Drug Administration). Bufadienolides (gamabufotalin, arenobufagin, hellebrigenin, telocinobufagin, bufotalin, cinobufotalin, bufalin, cinobufagin, resibufogenin) are the main composition of the remedy (>90%). CSI induced cell cycle arrest in G2/M phase of pancreatic cancer PANC-1 and MIA PaCa-2 cells, and induced their death through inhibiting the p-EGFR/KRAS/p-ERK1/2 signaling pathway. What is important, CSI was combined with erlotinib, an inhibitor of the epidermal growth factor receptor (EGFR) tyrosine kinase that is generally used in the treatment of several types of cancer [2]. The role of the EGFR mainly involves the regulation of epithelial tissue development and homeostasis. This protein is found on the surface of both normal and cancer cells and plays a role in cell growth and survival. In many cancers, the EGFR is overactive or mutated, causing uncontrolled cell growth and division [3]. In pancreatic cancer cells, erlotinib efficacy is reduced by KRAS mutations. However, the combination of erlotinib with CSI had a synergistic effect on cancer, both in vitro and in vivo. The therapy significantly decreased tumor weight and volume in tumor-bearing mice. This dual-agent treatment demonstrated greater therapeutic effectiveness in pancreatic cancer through inhibition of the erlotinib-induced resistance pathway involving KRAS and p-ERK1/2. 

Polyphenolic compounds are multifunctional bioactive agents that integrate anticancer, antioxidant, and anti-inflammatory activities. This combination makes them excellent candidates for the development of novel therapeutic strategies in modern medicine. Sahin et al. proved the role of curcumin, a naturally occurring plant compound, in preventing uterine fibroids—in this instance, leiomyoma was used in a hen model (Contribution 4). Daily curcumin consumption (up to 53 mg/kg) can significantly reduce the incidence of this type of tumor. As an antioxidant agent, curcumin induced nuclear factor E2-related factor 2 (Nrf2), which plays a role in enhancing antioxidant cell defense and protecting normal cells against carcinogens [4]. Furthermore, curcumin reduced the expression level of pro-inflammatory cytokines, namely IL-1β, IL-6, and TNF-α, in hen oviducts in a dose-dependent manner. This study showed that curcumin may be an effective agent in chemoprevention of one of the most frequently diagnosed gynecologic tumors in women.

In this Special Issue, one review presents the anticancer potential of *Sesbania* species, namely *S. grandiflora* (L.) Poir., *S. sesban* (L.) Merr., *S. cannabina* (Retz.) Poir. (Contribution 6), which are plants belonging to the Fabaceae family. These occur in tropical and subtropical regions of Africa, America, and Asia. Mokhtar et al. presented the results of the treatment of various cancer cells with extracts obtained from different plant parts of the *Sesbania* species, along with an evaluation of the mechanism of biological action. *Sesbania* species are rich in alkaloids, flavonoids, and saponins, which interact with molecular targets, including DNA and proteins involved in cell signaling pathways. Therefore, both extracts and individual secondary compounds have the potential to prevent the development of cancer. Mokhtar et al. also described the use of *Sesbania*-based silver nanoparticles that were coated with polyethylene glycol (PEG) as a delivery system, where active plant compounds were transported to specific target sites of cancer cells in a controlled manner. 

This Special Issue includes scientific papers demonstrating the significant role of plant extracts and their secondary metabolites in reducing inflammatory processes, fighting free radicals, preventing cancer development, and inhibiting tumor growth. We would like to thank all the authors who contributed to this Special Issue for sharing their research, which makes a significant contribution to the field of biological potential of plants.
